# High – but not standard-dose atorvastatin prevents the increase of plasma matrix metalloproteinase-2 triggered by acute coronary syndromes

**DOI:** 10.1097/MCA.0000000000001414

**Published:** 2024-09-19

**Authors:** Francesco Paciullo, Emanuela Falcinelli, Tiziana Fierro, Paolo Gresele, Maurizio Del Pinto

**Affiliations:** a Section of Internal and Cardiovascular Medicine, Department of Medicine and Surgery, University of Perugia; b Division of Cardiology, Perugia Hospital, Perugia, Italy

***To the editor,***

In patients with recent acute coronary syndrome (ACS), high-dose statins provide greater protection against death and major cardiovascular (CV) events compared with a standard-dose regimen. Apart from their lipid-lowering action statins exert pleiotropic effects that may contribute to reduce CV risk. Statins blunt inflammation, restore endothelial dysfunction, and reduce platelet and coagulation activation, thus preventing plaque destabilization and thrombus formation [1]. Statins modulate also the expression of some matrix metalloproteinases (MMPs), matrix-degrading enzymes that play a central role in atherogenesis and plaque rupture [1,2]. MMP-2 is particularly abundant in atherosclerotic plaques where it plays a role in their unstabilization and, when released, it triggers platelet activation and thrombus formation [3]. Circulating MMP-2 rapidly increases soon after an ACS and it represents a biomarker of plaque instability and a predictor of poor outcome [2,4].

The aim of the present proof-of-concept study was to evaluate the effect of a standard- or high-dose atorvastatin on the acute changes in plasma MMP-2 triggered by ACS.

Patients with a diagnosis of unstable angina/non-ST-elevation myocardial infarction (based on cardiac ultrasound imaging and at least two among the following: typical chest pain or angina equivalents; serial changes in 12-lead ECG; significant myocardial biomarkers variation), were enrolled. The study was conducted before the publication of the latest guidelines for dyslipidemia management thus allowing treatments to include those accepted at the time of study planning.

Patients were divided into three groups: standard-dose atorvastatin, consisting of 20 mg/day started immediately after hospitalization, high-dose atorvastatin, consisting of 80 mg/day started immediately after hospitalization, and no-statin, consisting in the use of 20 mg/day atorvastatin started 96 h after hospitalization. The statin regimen was decided by the caring cardiologist based on the patient’s lipid levels and CV risk profile. A group of age- and sex-matched healthy controls not treated with statins was studied in parallel.

Peripheral blood was obtained on the day of hospitalization, 3 and 5 days later. MMP-2 and tissue inhibitor of matrix metalloproteinases-2 (TIMP-2) were measured in plasma by zymography [3] or by ELISA (Thermo Fisher Scientific, Waltham, Massachusetts, USA) [4], respectively.

Considering that atorvastatin was previously shown to reduce serum MMP-2 by 40% in patients with heart failure, we calculated that it would be necessary to enroll 14 subjects per group, with a 95% confidence margin and 1% margin of error, to show a significant difference among statin regimens. Differences between groups were estimated by one-way analysis of variance using the GraphPad Prism 8.4 for Windows (GraphPad Software 225 Franklin Street. Fl. 26 Boston, Massachusetts).

A total of 40 consecutive unstable angina/non-ST-elevation myocardial infarction patients (13 high-dose atorvastatin, 14 standard-dose, and 13 no-statins) and 30 age- and sex-matched healthy controls were enrolled (Table 1).

**Table 1:** Demographics and clinical characteristics of the study groups at enrollment

	High-dose group (*n* = 13)	Standard-dose group (*n* = 14)	No-statin group (*n* = 13)	HC (*n* = 30)
Age, years	67 ± 2	70 ± 3	69 ± 3	65 ± 8
Female, *n* (%)	5 (38)	2 (14)	2 (15)	8 (26)
Occluded coronary vessels (*n*)	1.2 ± 0.3	1.5 ± 0.3	0.7 ± 0.2	NA
Time from the event (h)	20 ± 8	24 ± 6	11 ± 7	NA
Troponin (ng/ml)	6.1 ± 2.6	8.1 ± 2.7	3.1 ± 0.5	NA
CPK-MB (ng/ml)	35.3 ± 11	37.5 ± 15	24 ± 5	NA
Leukocytes (×10^3^/μl)	9.8 ± 1.3	9.4 ± 1.0	7.1 ± 0.6	5.5 ± 0.3
Platelets (×10^3^/μl)	223 ± 27	218.7 ± 17	192.3 ± 32	220 ± 15.7
Total cholesterol (mg/dl)	195 ± 15	226 ± 33	177 ± 16	156 ± 7
LDL (mg/dl)	124 ± 12	156 ± 25	109 ± 16	114 ± 8
HDL (mg/dl)	39 ± 1.4	47 ± 7	44 ± 4	45 ± 3.5
Triglycerides (mg/dl)	160 ± 24	121 ± 13	115 ± 18	126 ± 4
Comorbidities, *n* (%)				
Hypertension	4 (30.7)	4 (28.5)	4 (30.7)	3 (10)
Type 2 diabetes	0 (0)	0 (0)	3 (23)*	0 (0)
Obesity	0 (0)	2 (14.2)*	1 (7.6)	0 (0)
Smoke	4 (30.7)†	3 (21.4)	0 (0)	5 (16.6)
Hypercholesterolemia	3 (23)	2 (14.2)	0 (0)	2 (6.6)
Previous CAD	2 (15.3)*	2 (14.2)	1 (7.6)	0 (0)
Medications during hospitalization, *n* (%)				
Aspirin	13 (100)	14 (100)	13 (100)	NA
P2Y_12_ inhibitors	11 (84)	13 (92)	13 (100)	NA
ACE inhibitors/β-blockers	10 (77)	14 (100)	13 (100)	NA

Data are presented as mean ± SEM.

ACE, angiotensin-converting enzyme; CAD, coronary artery disease; CPK-MB, creatine phosphokinase-MB; HC, healthy controls; HDL, high-density lipoprotein; LDL, low-density lipoprotein; NA, not applicable.

* *P* < 0.05 vs HC.

† *P* < 0.05 vs no-statin.

Other comparisons: *P* = NS.

Patients in the no-statin group showed an increase of plasma MMP-2 from baseline to day 3 after ACS (+102.8 ± 6 ng/ml), in the standard-dose statin groups plasma MMP-2 increased less and not significantly (+52 ± 28 ng/ml), while in the high-dose atorvastatin group, a significant reduction was observed (Fig. 1a–c).

**Fig. 1:**
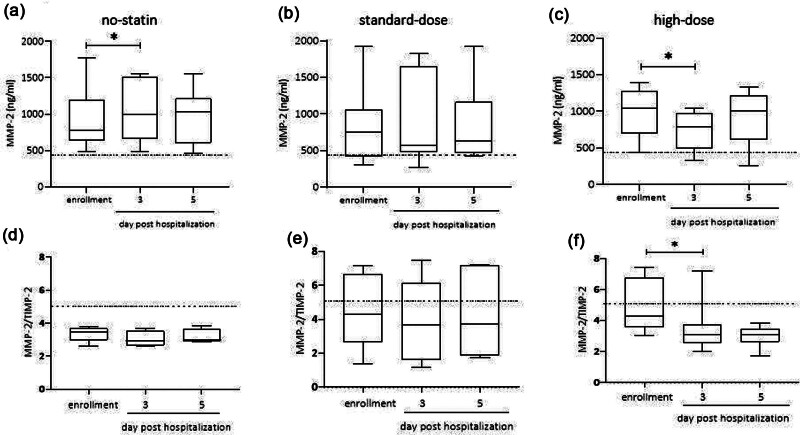
Time course of plasma MMP-2 (a–c) and MMP-2/TIMP-2 ratio (d and e) at hospitalization and 3 and 5 days after in subjects treated with different statin regimens (medians and interquartile ranges). Dotted line represents median levels in healthy controls. MMP-2, matrix metalloproteinase-2; TIMP-2, tissue inhibitor of matrix metalloproteinases-2. *P < 0.05 vs enrollment.

The MMP-2/TIMP-2 ratios did not change in the no-statin and in the standard-dose groups, while they decreased significantly in the high-dose atorvastatin group at day 3 (Fig. 1d and e).

Our study shows that high – but not standard-dose atorvastatin started immediately after an ACS significantly blunts the early rise of circulating MMP-2. Interestingly, this effect was evident very early after treatment initiation, probably earlier than the hypolipemic effect which takes at least 4 weeks to develop, similar to other pleiotropic actions of statins [1].

MMP-2 activity is tightly controlled by TIMP-2, and an alteration of the balance between MMP-2 and TIMP-2 in favor of the latter results in reduced MMP-2 activity [4]. We show here that a high-dose statin regimen significantly reduces the MMP-2/TIMP-2 ratio, an index of MMP-2 activity [4], further strengthening the significance of our observation.

Given that raised circulating MMP-2 soon after an ACS is considered a CV risk biomarker [2,5] our observation may contribute to explain the early protective effects of high-dose statins against recurrent CV events.

Our study has some limitations, including the low sample size, however, the prospective design, sample size calculation beforehand, and the good comparability of the treatment groups should counterbalance this potential bias; the lack of correlation with clinical outcome, however, the incidence of recurrent CV events in ACS patients under optimal treatment within the first 5 days is low, compatible with the lack of events in our investigation; the lack of randomization to the treatment groups raising the risk of selection bias: however, inclusion criteria were rigorously predefined and the careful selection of cases and controls made the study populations very comparable.

In conclusion, our study shows that high – but not standard-dose atorvastatin started immediately at hospitalization significantly reduces the rise of circulating MMP-2 associated with ACS, a phenomenon possibly contributing to the protective effects of high-dose statins against adverse CV outcomes. These results need to be confirmed in larger prospective studies correlating the statin-induced blunting of MMP-2 rise after an ACS with long-term CV events.

## Conflicts of interest

There are no conflicts of interest.
